# Clinical metabolomics investigation of rheumatoid arthritis patients receiving ayurvedic whole system intervention

**DOI:** 10.1016/j.jaim.2024.101009

**Published:** 2024-07-06

**Authors:** Sanjeev Rastogi, Ankita Verma, Rimjhim Trivedi, Anuj Shukla, Dinesh Kumar

**Affiliations:** a Ayurveda –Arthritis Treatment and Advanced Research Center (A-ATARC), Department of Kaya Chikitsa, State Ayurvedibc College and Hospital, Lucknow University, Lucknow, 226003, India; b Centre of Biomedical Research (CBMR), SGPGIMS Campus, Lucknow, 226014, Uttar Pradesh, India; c Academy of Scientific and Innovative Research (AcSIR), Ghaziabad, Uttar Pradesh, 201002, India

**Keywords:** Rheumatoid arthritis, Ayurveda whole system intervention, Amavata, NMR based clinical metabolomics, Metabolic biomarkers, Treatment response

## Abstract

**Background:**

Arthritis is a common clinical condition seen in Ayurveda clinics. Clinical trials have reported Ayurvedic interventions to be of benefits in many arthritic conditions including Rheumatoid Arthritis (RA). No mechanistic details however are available about how such interventions on their own or as a combination of whole system Ayurveda might be working. **Objective:** The study aims to evaluate simultaneously the clinical outcome of Ayurveda whole system (AWS) intervention in RA patients and identifying the serum metabolic signatures which could be useful for diagnosing the disease and monitoring treatment response.

**Material and methods:**

RA patients (n = 37) simultaneously diagnosed as *Amavata* fulfilling the specific inclusion and exclusion criteria were recruited in the study and were given Ayurveda whole system (AWS) intervention comprised of oral medicines, local therapy and dietary recommendation for 3 months. The clinical and serum metabolic changes were investigated for pre-treatment RA patients (baseline RA group, n = 37) and post-treatment RA patients (following treatment of 6-weeks (RA_F, n = 26) and three months (RA_T, n = 36). For comparative serum metabolomics analysis, 57 normal healthy control (HC) subjects were also involved and the serum metabolic profiles were measured at high-field 800 MHz NMR spectrometer. The serum metabolic profiles were compared using multivariate statistical analysis and discriminatory metabolic features were evaluated for diagnostic potential using receiver operating characteristic (ROC) curve analysis.

**Results:**

A significant reduction in DAS-28 ESR, AAM Score, total swollen joints, total tender joints were observed following AWS intervention. The clinical outcomes were concordant with changes in metabolic profiles of RA patients as these were also shifting towards the normal levels following the intervention. Compared to healthy control (HC) subjects, the sera of baseline RA patients were characterised by increased circulatory level of succinate, lysine, mannose, creatine, and 3-Hydroxybutyrate (3-HB) and decreased levels of alanine. The present study also evaluated the serum metabolic ratios for their discriminatory and diagnostic potential and notably, six metabolic ratios (KHR, KThR, KVR, GHR, PTR and SHR) were found significantly altered (elevated) in baseline RA patients. However, in RA patients receiving AWS treatment, these metabolic changes showed marked convergence towards the metabolic signatures of healthy controls.

**Conclusion:**

This first of its kind study clearly shows the clinical efficacy of Ayurvedic Whole System (AWS) intervention in the management of Rheumatoid Arthritis (RA), as demonstrated by significant improvements in key clinical parameters. The intervention not only alleviated symptoms but also induced a profound metabolic shifting towards normalization; thus, underscoring the potential of AWS intervention to modulate cellular metabolism in a manner that facilitates a return to homeostasis in RA patients. However, future studies are imperative to confirm these preliminary observations and delineate the underlying mechanisms of action of intervention in cases of RA.

## 1 Introduction

Arthritis is a highly prevalent clinical condition in Ayurveda clinics across the country [1]. Over 80% of all suffering with musculoskeletal disorders in India have reported to use some form of complementary or alternative medicine at some point of time of their illness [2]. Of these CAM therapies, Ayurveda is reported to be chosen by highest proportion of the people. Reported reason for choosing Ayurveda or other alternative therapies are found related to inadequate pain relief through conventional medicine. A survey at a secondary care hospital of Ayurveda, identified similar reason behind the choice of Ayurveda. It is also the direct or indirect evidences of its effectiveness, which helped people to make their decision about choice of a particular system of health care in a particular type of disease [3]**.**

The commonest arthritis seen in Ayurveda clinics is osteoarthritis followed by rheumatoid arthritis (RA) and gouty arthritis. Spondylo arthropathy is also one common clinical entity available in Ayurveda clinics. Seeing the huge potential of Ayurveda interventions in arthritis management by looking at substantial input of such patients in Ayurveda clinics, and seeing that it has its positive and stable clinical impacts as is often self-reported by the patients [4], this was often argued to look at what might be the mechanistic insight of Ayurvedic interventions in such conditions. Black-box design is recommended for clinical trials related to traditional medicines including Ayurveda where more than one modality of the intervention is involved and where it is highly difficult to make a judgement about which component of the intervention is actually working. Since from the traditional perspective, every component of the treatment protocol may have an equal importance, this is impractical for a traditional practitioner to choose some part of the intervention over the other and to evaluate their fractional efficacy. This is also argued that Ayurvedic interventions are similar to placebo and they offer only symptomatic improvements till the actual intervention is offered [5]**.** Clinical improvements seen in the Ayurvedic clinics are often counter argued to be the subjective observations having high variability. In the absence of robust double blind clinical studies, such claims are often disrespected and disregarded. Unfortunately doing controlled and blinded randomised trials in Ayurveda has its own set of problems related to masking and ethics [6,7]. Large dosage forms of Ayurvedic formulations diversity of formulations like avaleha and asva/arisha does not have a suitable placebo. Multiple dosage forms, multiple interventions intertwined with dietary and life style advices makes it further complex to effectively organise a Randomized controlled trial in Ayurveda. In such conditions, metabolomic studies have come up as a great help to establish the effects of Ayurvedic interventions by looking into the precise metabolomic changes occurring in the patients and comparing them with the corresponding clinical changes observed post intervention [8]. Such observation is supposed to bring a great thrust to the Ayurvedic concept of *samprapti vighatana* or dissociation of pathogenesis by seeing that the metabolomic profiling is shifting towards normal among the people who are receiving Ayurvedic interventions.

This study was conducted to observe the clinical and metabolomic changes observed in a pool of *amavata* patients who were simultaneously diagnosed as RA and registered in the study following a strict inclusion and exclusion criteria. All the registered patients were evaluated for their metabolomics profile initially, given the standard AWS intervention for three month and subsequently evaluated again to observe changes in the metabolomic profile. The metabolomic observations made in the study were found to be in tune to the clinical changes observed and were suggestive of fine metabolomic changes at the cellular level among the people suffering with RA and receiving Ayurveda interventions.

## 2 Material and Methods

### 2.1 Ethical approval and subject Recruitment

The study protocol was approved by the Institutional Ethics Committee, State Ayurvedic College and Hospital, Lucknow, Uttar Pradesh, India (Ref No. SAC/IEC/2021/34; Dated: September 17, 2021 and was registered in CTRI (Registration No. CTRI/2022/07/044,348). The study included serum samples collected from: RA Patients (RA), Follow Up (RAF), Post Treatment (RAT) and Healthy Control (HC). As per approval, the study enrolled RA patients diagnosed as per the ACR 2010 criteria and *Amavata* diagnosed on the basis of classical *Amavata* symptoms as depicted in Madhava Nidana and as elaborated by Rastogi et al., 2009 [9,10] and met specific inclusion and exclusion criteria. The enrolled patients received AWS intervention (which includes oral medicines, local therapy, and dietary recommendations) at the Ayurveda – Arthritis Treatment and Advanced Research Centre, State Ayurvedic College and Hospital, Lucknow, India (A-ATARC OPD at SAC & Hospital, Lucknow) for 3 Months. The study's control group comprised healthy volunteers (apparently healthy on clinical and laboratory evaluations) with age and gender proximity, devoid of any underlying diseases or medication intake, ensuring a robust comparison framework.

### 2.2 Diagnosis criterion and AWS intervention

RA was diagnosed as per the ACR 2010 criteria [11]. *Amavata* was diagnosed on the basis of classical *Amavata* symptoms as are depicted in *Madhava Nidana* and are elaborated by Rastogi et al., 2009 [10].

#### 2.2.1 Inclusion criteria

• Patients simultaneously diagnosable as RA and Amavata on the basis of their respective diagnostic criteria.
• Patients of either gender between age 20–60 years (both included)
• Patients not having any other systemic illness
• Patients not in remission state as per DAS-28 ESR criteria.
• Patients not having joint deformities.
• Patients having given consent to participate in the study
• Patients who have not been on any ayurvedic therapy for amavata in past 3 months.
• Patients who have observed a wash out period of 10 days before the initiation of ayurvedic therapy.

#### 2.2.2 Exclusion criteria

• Patients having any known allergy to any ayurvedic intervention.
• Patients having severe disease activity with joint destruction and joint deformity.
• Patients having co morbid joint conditions like Osteoporosis, Gout, and Osteoarthritis etc.
• Patients having other autoimmune conditions.
• Patients requiring urgent care.
• Patients unable to follow comprehensive treatment program recommended by Ayurveda.

Inclusion and exclusion criteria for healthy controls was limited to similar age (20–60 years of either gender) and apparently not having any disease or any ongoing medication as revealed by the history. World Health Organization (WHO) considers traditional medicine as a holistic system or whole system intervention [12]. The Whole System Research (WSR) promotes use of “personalized intervention” designed by the physician on case-to-case basis considering disease state. WHO recommended whole system intervention in field of traditional medicine research implies that research efforts in traditional medicine should not focus solely on isolated components or individual interventions but should instead consider the entire traditional medicine system as a cohesive entity [13]. Therefore, WHO's recommendation for whole system intervention underscores the importance of taking a holistic and integrative approach to traditional medicine research which involves studying how different components of traditional medicine interact with each other and with conventional medical treatments, as well as assessing their effectiveness, safety, and cultural relevance. Within this framework, the Ayurvedic whole system intervention in standard form used in this study (Table 1) includes all pharmacological and nonpharmacological components (*Nidana Parivarjana, Ahara Vyavastha, Vihara Vyavastha, Aushadhi Vyavastha*) [[14], [15], [16]].

**Table 1:** Whole System Ayurveda Recommendation for RA/Amavata patients.

**Dietary Recommendations** • Absolute avoidance of curd, cold water & direct cold air of Fan/Cooler/AC. • Drink warm water. • Note down & avoid all food item that aggravates their disease individually. (Common food items noticed by majority of patients at ATARC are Brinjal, Ladyfinger, Jackfruit, Watermelon, Mango etc.) • Avoid fruit that are very sweet and watery in nature like Watermelon, Muskmelon etc. • Avoid excessive oily food or the food that are not easily digested. • Avoid intake of Naveen anna (new grain), • Avoid urad dal & can take pulses like moong dal etc. **Life style recommendations** • Avoid Swimming and all water sports. • Try to use warm water for bathing/showering. • Try to always use protective warm clothing during cold weather. • Try to avoid travelling in cold/rainy weather. • Daily mild exercise according to vyayamama shakti & Sunbathing. • Follow an active lifestyle or join any activities group. • Understand the nature of your disease and make necessary changes in your daily life style from now on. • Listen and follow your doctor's advice carefully. • Take your medication on time regularly. • Have patience because Ayurvedic medication take time for accumulation of desired dose in the body to show adequate visible responses. **Pharmacological interventions** • Rasaushadhi (125 mg BD): Amavatarirasa • Vati (1 BD): Chitrakadi Vati/Sanjeeni Vati/Agnitundi Vati • Kwath (30 ml BD): Dashmool kwatha • Choorna (5 mg BD): Hingvashtak/Panchakol/Ajmodadi/Vaisvanar choorna • Guggulu (1 BD): Sinhanada/Yogaraja/Shigru guggulu • Avaleha (1 TSF BD): Amritabhalataka • Taila: Eranda taila (5 ml HS), • Bahaya Chikitsa: Swedana- Saindhava Baluka Sweda (SBS), • Snehana- Saindhavadi taila/Mahavishgarbha taila.

### 2.3 Sample collection and processing for metabolomics study

The blood samples were collected from 126 subjects (RA = 37, RAF = 26, RAT = 36 and HC = 27) in a plain red topped serum glass tubes. Approximately 2 mL of blood was drawn from each subject and kept undisturbed at room temperature (23–30 °C) for 30–40 min allowing it to clot and centrifuged at 3000 RPM (rotation per minute) for 10 min. The supernatant was collected and stored at −80 °C temperature for metabolomic study.

For NMR studies, the stored samples were thawed at room temperature prepared by vortexed and 300 μL of serum was mixed with 300 μL of sodium phosphate buffer (Buffer strength 50 mM:0.9% saline prepared in 100% Deuterium Oxide) containing the known concentration of 0.2 mM TSP (Sodium salt of 3-trimethylsilyl-(2,2,3,3-*d*4)-propionic acid for the purpose of chemical shift referencing) (Sigma, Rhode Island, USA). The mixture was centrifuged at 16,278×*g* for 5 min and 550 μL of each sample was transferred to 5 mm NMR tube (Wilmad Glass, USA).

### 2.4 Nuclear magnetic resonance (NMR) spectroscopy measurements and assignment

The NMR spectra was acquired using the 1D ^1^H CPMG (Carr–Purcell–Meiboom–Gill) pulse program at 300 K using the 800 MHz NMR spectrometer (Bruker, Avance III, equipped with Cryoprobe) at Centre of Biomedical Research, Lucknow. The CPMG NMR spectra were initially calibrated by referencing the singlet peak of TSP at 0.0 (ppm) and then manually adjusted for phase and baseline distortions using Bruker software (Topspin 3.6, Academic version). Subsequently, the processed NMR spectra (format 1rr file) were imported into the PROCESSOR module of the commercial software Chenomx (NMR Suite, v9.0, Chenomx Inc., Edmonton, Canada). To mitigate analytical variations arising from sample dilution, each spectrum was internally referenced to the endogenous metabolite formate δ (8.43) ppm, with formate concentration set to 30 μM, as previously reported in human blood plasma/serum. Given the significant impact of baseline on NMR spectrum quantification, each spectrum underwent further refinement for baseline correction using the Whittaker spline method available within the Chenomx software suite. The metabolite resonances in 1D ^1^H CPMG NMR spectra were assigned and quantified using Profiler module of Chenomx NMR Suite (containing 800 MHz chemical shift database). The metabolic concentration was exported in CSV format (comma-separated values) for further analysis. Multivariate analysis was performed using SIMCA-P+ software (version 14.1, MKS Umetrics, AB).

### 2.5 Statistical analysis

Univariate statistical analysis was conducted utilizing Metaboanalyst (https://www.metaboanalyst.ca/), a free web-based server built on the R programming language [17]. Differences in circulatory metabolic concentrations between groups were assessed using the Student t-test analysis method and visualized through Box and Whisker plots. The diagnostic capacity of altered Metabolites was assessed through Receiver Operating Characteristic (ROC) curve analysis [18]. A metabolite with a value greater than 0.85 for the Area under the ROC curve can be considered deemed as diagnostic candidate. The Biomarker module of Web-based software ‘Metaboanalyst v5.0’ was used for screening metabolic ratios of diagnostic potential. For this, the top 20 ratios of diagnostic potential were first identified employing inbuilt function of Biomarker module.

## 3 Observation and results

**Fig. 1:**
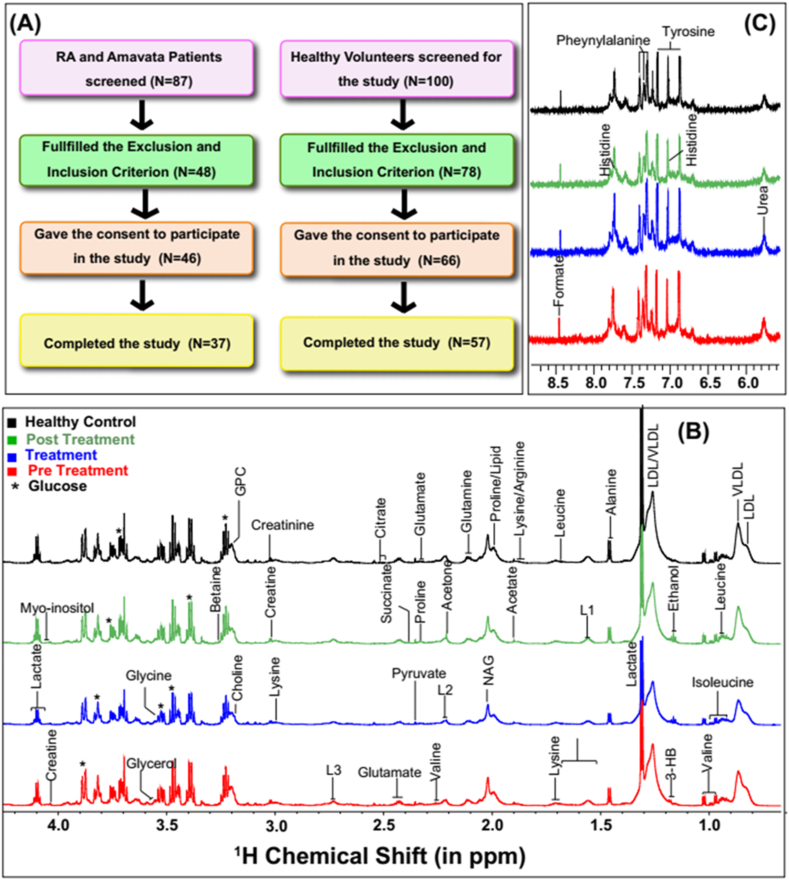
(A) Study Flow Chart for RA and Amavata Patients (left) and study Flow Chart for healthy volunteers (right). (B,C) The cumulative 1D ^1^H CPMG NMR spectra of serum samples obtained from healthy Control (HC, in Black), Pre Treatment (in Red), 6-week follow-up patients (RA_F, in blue) and following three months of treatment (RA_T, in green). The spectral regions shown in B and C correspond to δ (0.8–4.25) and B. δ (5.4–8.7) and the spectra in panel B are magnified sixteen times as compared to spectra in panel A for better clarity.

Of 87 cases initially screened, 37 finally completed the study hence included in the analysis (30 Female and 7 Male). Of 100 healthy volunteers screened for the study 66 initially gave their consent to participate but finally only 57 (13 Male and 44 Female) could complete the study and hence were included in the analysis. The study flow chart is shown in Fig. 1A. Average age of study participants in the intervention group was 43.35 years and in the healthy control group was 43.56 years. This is apparent that there is a close resemblance in the age group of patients receiving the intervention and healthy controls.

### 3.1 Clinical observations

#### 3.1.1 Ama assessment

All study participants in the intervention group were observed for the AMA score which is presumed to be an Ayurvedic marker for amavata disease activity [19]. Mean Ama score in the RA patients was observed to be 63.84 ± 13.91 before treatment which was reduced to 33.19 ± 13.84 after 3 month of Ayurveda intervention. The mean change in ama score was found to be 48.01% which was highly significant (Table 2). Participants' evaluations of the Tender Joint Count (TJC) score were measured using mean scores, standard deviation (SD), percentage change, z-values, and p-values. During the post-treatment phase, participants' TJC score reduced from 17.11 to 11.70 having a percentage change of 31.60% which was highly significant (p < 0.001) (Table 3).

**Table 2:** Changes in AMA Score evaluated in patients before (pre) and after (post) the treatment.

Timeline	AMA SCORE			WILCOXON TEST	
	MEAN	SD	% CHANGE	Z-VALUE	P-VALUE
**PRE (Base line)**	63.84	13.91	NA	NA	NA
**FOLLOW UP (6 weeks)**	45.41	13.42	28.87	−5.31	<0.001
**POST (3 months)**	33.19	13.84	48.01	−5.30	<0.001

**Table 3:** Changes in Tender Joint Count (TJC) Score (28 Joints) evaluated in patients before (pre) and after (post) the treatment.

TJC (Tender joint count)	Score			Wilcoxon test	
	Mean	SD	% change	z-value	p-value
Pre test (Base line)	17.11	5.57	NA	NA	NA
Follow up (6 weeks)	13.84	5.05	19.12	−4.25	<0.001
Post test (3 months)	11.70	5.07	31.60	−4.31	<0.001

Participants' assessments of the Swollen Joint Count (SJC) score were measured using mean scores, standard deviation (SD), percentage change, z-values, and p-values. In the pre-test phase, participants reported an average SJC score of 6.27, accompanied by a standard deviation of 4.19 (Table 4). Following the follow-up interval, the average SJC score notably decreased to 4.78, with a standard deviation of 3.53. This signified a considerable percentage change of 23.71%. The Wilcoxon test yielded a z-value of −3.62 and a p-value of less than 0.001, indicating a statistically significant reduction in the SJC score following the follow-up period (Table 4). During the post-test phase, participants' SJC score further diminished, reflecting an average score of 3.43 and a standard deviation of 3.68. This translated to a substantial percentage change of 45.26%. The Wilcoxon test resulted in a z-value of −4.56 and a p-value of less than 0.001, reaffirming the statistically significant reduction in the SJC score following the intervention (Table 4).

**Table 4:** Changes in SJC Score (28 joints) evaluated in patients before (pre) and after (post) the treatment.

SJC (Swollen joint count)	Score			Wilcoxon test	
	Mean	SD	% change	z-value	p-value
Pre test (base line)	6.27	4.19	NA	NA	NA
Follow up (6 weeks)	4.78	3.53	23.71	−3.62	<0.001
Post test(3 months)	3.43	3.68	45.26	−4.56	<0.001

Participants' assessments of the ESR were quantified using mean scores, standard deviation (SD), percentage change, t-values, and p-values. In the pre-test phase, participants reported an average ESR of 39.46, with a standard deviation of 21.92 (Table 5). Following the follow-up interval, the average ESR decreased to 35.41, accompanied by a standard deviation of 14.92. This signified a percentage change of 10.27%. The paired *t*-test yielded a t-value of 1.93 and a p-value of 0.061, indicating a notable reduction in ESR following the follow-up period, although statistical significance was not achieved (Table 5). During the post-test phase, participants' ESR score increased to 39.22, reflecting a standard deviation of 21.23. This translated to a minimal percentage change of 0.62%. The paired *t*-test resulted in a t-value of 0.06 and a p-value of 0.953, highlighting that the change in ESR was not statistically significant following the intervention (Table 5).

**Table 5:** Changes in circulatory ESR levels evaluated in patients before (pre) and after (post) the treatment.

ESR	Score			Paired *t*-test	
	Mean	SD	% change	t-value	p-value
Pre test (Base line)	39.46	21.92	NA	NA	NA
Follow up (6 weeks)	35.41	14.92	10.27	1.93	0.061
Post test (3 months)	39.22	21.23	0.62	0.06	0.953

Participants' evaluations of the PGHA score were quantified using mean scores, standard deviation (SD), percentage change, z-values, and p-values. Following the follow-up interval, the average PGHA score decreased to 7.00, accompanied by a standard deviation of 1.70. This signified a notable percentage change of 14.24% (Table 6). The Wilcoxon test yielded a z-value of −4.36 and a p-value of less than 0.001, indicating a statistically significant reduction in the PGHA score following the follow-up period. During the post-test phase, participants' PGHA score continued to decrease, reflecting an average score of 6.68 and a standard deviation of 1.65 (Table 6). This translated to a substantial percentage change of 18.21%. The Wilcoxon test produced a z-value of −4.06 and a p-value of less than 0.001, underscoring the statistically significant reduction in the PGHA score following the intervention (Table 6).

**Table 6:** Changes in PATIENT GLOBAL HEALTH ASSESMENT (PGHA) Score evaluated in patients before (pre) and after (post) the treatment.

PGHA (PATIENT GLOBAL HEALTH ASSESMENT)	Score			Wilcoxon test	
	Mean	SD	% change	z-value	p-value
Pre test (Base line)	8.16	1.74	NA	NA	NA
Follow up (6 weeks)	7.00	1.70	14.24	−4.36	<0.001
Post test (3 Months)	6.68	1.65	18.21	−4.06	<0.001

Participants' assessments of the DAS28-ESR score were measured using mean scores, standard deviation (SD), percentage change, z-values, and p-values (Table 7). In the pre-test phase, participants reported an average DAS28-ESR score of 6.54, accompanied by a standard deviation of 0.64. As this marked the baseline measurement, the percentage change is indicated as “NA” (Not Applicable), and no z-value or p-value was calculated (Table 7). Following the follow-up interval, the average DAS28-ESR score decreased to 6.06, with a standard deviation of 0.65. This signified a notable percentage change of 7.35%. The Wilcoxon test yielded a z-value of −4.78 and a p-value of less than 0.001, indicating a statistically significant reduction in the DAS28-ESR score following the follow-up period. During the post-test phase, participants' DAS28-ESR score continued to decrease, reflecting an average score of 5.67 and a standard deviation of 0.72. This translated to a substantial percentage change of 13.32%. The Wilcoxon test produced a z-value of −4.91 and a p-value of less than 0.001, reaffirming the statistically significant reduction in the DAS28-ESR score following the intervention (Table 7).

**Table 7:** Changes in **DAS28-ESR** Score evaluated in patients before (pre) and after (post) the treatment.

DAS28-ESR Score	Score			Wilcoxon test	
	Mean	SD	% change	z-value	p-value
Pre test (base line)	6.54	0.64	NA	NA	NA
Follow up (6 weeks)	6.06	0.65	7.35	−4.78	**<0.001**
Post test (3 months)	5.67	0.72	13.32	−4.91	**<0.001**

#### 3.1.2 Metabolic disturbances associated with rheumatoid arthritis (RA)

**Fig. 2:**
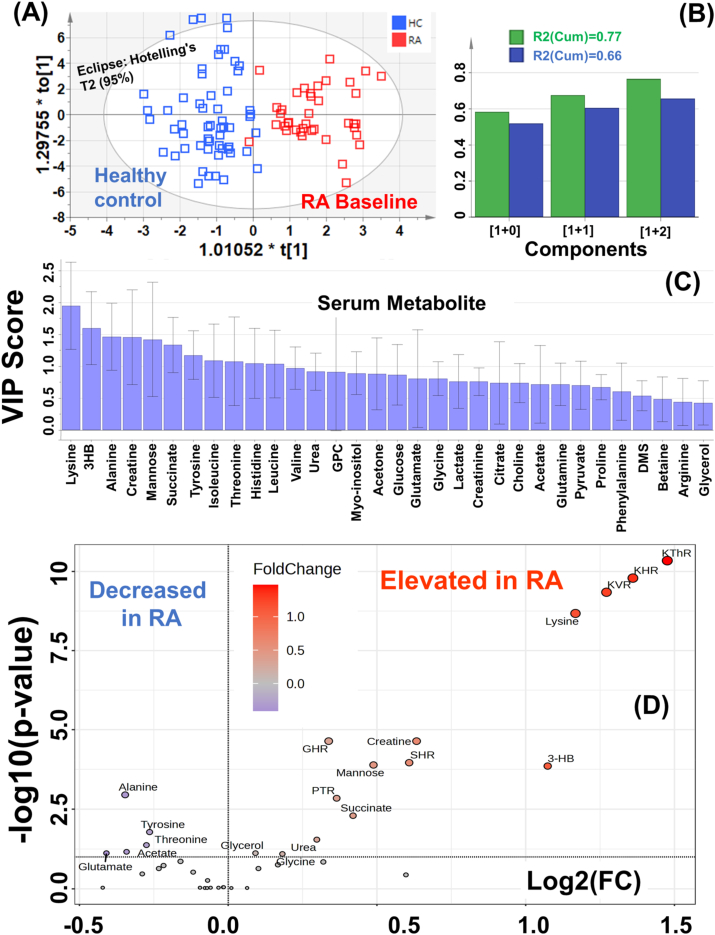
OPLS-DA prediction analysis results based on 32 serum metabolic features. **(A)** OPLS-DA score plot showing serum metabolic disparity between study groups: RA (n = 37) and healthy control (HC,n = 57). **(B)** The validation of OPLS-DA model (R2(Cum) = 0.77; Q2(Cum) = 0.66). **(C)** VIP score plot derived from OPLS-DA analysis in SIMCA highlighting the metabolites of discriminatory significance. **(D)** Volcano plot reporting p values against fold change (FC). The volcano plot indicates −log 10 (p value) for serum metabolic profiles (Y-axis) plotted against their respective log2(FC) (X-axis). The explicit fold change (FC) values can be calculated following the mathematical process; FC: is real number, FC > 0; log2(FC) = y and FC = 2^y^ (see the explicit FC values in Table 8).

The cumulative NMR spectra of four study groups are stacked in Fig. 1B for comparative evaluation. The visual inspection showed that the serum metabolic profiles are apparently different in RA patients compared to HC subjects. The present study aims to evaluate the intervention effect of Whole system ayurvedic treatment following serum metabolomics approach based on analysis of 1D ^1^H NMR spectra recorded on the serum samples of RA patients before intervention (RA; n = 37), during the intervention (RAF; n = 26) and post intervention (RAT; n = 36) with reference to Healthy Control (HC; n = 27). Before starting the concentration profiling of serum metabolites using NMR suite of CHENOMX software, first the CPMG spectral peaks were assigned for specific serum metabolites, making composite use of the 800 MHz metabolite spectral database library of CHENOMX NMR suite and previously reported NMR chemical shifts of serum metabolites [[20], [21], [22], [23]] (Fig. 1B). Next, the profiler module of NMR suite of CHENOMX software was used to estimate the concentration levels of 32 metabolite with respect to internal reference formate [24]: 3-hydroxybutyrate (3-HB), acetate, acetone, alanine, arginine, betaine, choline, citrate, creatine, creatinine, dimethyl-sulfone (DMS), glucose, glutamate, glutamine, glycerol, glycine, isoleucine, lactate, leucine, lysine, mannose, phenylalanine, proline, pyruvate, succinate, threonine, tyrosine, urea, valine, myo-Inositol, glycerophosphocholoine (GPC), and histidine. The estimated concentration profiling data (in the “CSV” file format in an excel sheet) was used for univariate and multivariate analysis. First, 32 serum metabolic profiles were compared between the HC and baseline RA subjects using multivariate analysis method followed by statistical analysis. These two study groups were compared for metabolic disparity using supervised orthogonal partial least square discriminant analysis (OPLS-DA, See Fig. 2).

The 2D score plot clearly revealed that the serum samples of RA and HC were well separated and clustered thus indicating that the metabolomics profiles of two groups are distinctively different (Fig. 2A). The OPLS-DA model cross-validation parameters, R2 (explained variation) and Q2 (predictive capability) were significant (R2>0.77, Q2>0.66, Fig. 2B) suggests that discriminatory model possesses a satisfactory fit with good predictive power. The discriminatory metabolic entities were identified based on VIP (Variable Importance in Projection) score plot (Fig. 2C) and further evaluated for statistical significance (p value < 0.05). To be mentioned here is that VIP score plot serves as a navigational tool for prioritizing or screening the most relevant features through indexing of variables based on their discriminatory significance between different study groups. Variables with higher VIP scores are indicative of greater discriminatory power, hinting their potential as biomarkers or key targets for further statistical testing or validation. This is particularly vital in metabolomics studies involving hundreds of metabolic features, where the sheer volume of data can be overwhelming without proper prioritization. However, the present study involves only 32 metabolic features, therefore, the VIP score plot shown in Fig. 2C can be considered as a guiding graph to pinpoint the most influential features that differentiate baseline RA patients from HC subjects. Considering the VIP score cut-off value of 0.8, nineteen metabolic features of discriminatory significance were identified: lysine, 3-hydroxybutyrate, alanine, creatine, mannose, succinate, tyrosine, isoleucine, threonine, histidine, leucine, valine, urea, glycerophosphocholine (GPC), myoinositol, acetone, glucose, glutamate and glycine. Further, these discriminatory metabolic features were tested for their statistical significance and fold change parameters using volcano plot method shown in Fig. 2D. Compared to HC subjects, the sera of RA patients were characterized by (a) elevated circulatory levels of Lysine, 3-Hydroxybutyrate (3HB), mannose, creatine, succinate, urea, glycine and glycerol and (b) decreased levels of alanine, tyrosine, threonine, glutamate and acetate (Fig. 2D). Note, the horizontal axis in volcano plot (Fig. 2D) represents log2(FC). Therefore, the fold change (FC) values (calculated following the mathematical process; FC: is real number, FC > 0; log2(FC) = y and FC = 2^y^) are listed Table 8.

**Table 8:** Receiver operating characteristic (ROC) curve analysis performed for 39 serum metabolic features (32 metabolic entities and 7 metabolic ratios). The calculated area under the ROC (AUC) curve values are listed to highlight the diagnostic potential of metabolites. The symbol asterisk “*” represents the metabolic change is statistically significant, where *, **, ***, and **** represent the metabolic change with p-value <0.05 < 0.001, <0.0001 and < 0.00001. The columns six and seven shows the quantitative fold change between the metabolic levels and the last column shows the relative change in metabolic level in the baseline RA patients.

#	Metabolite	AUC	p-value	Level of Statistical significance	Fold Change (FC)	Log2(FC)	Relative Change in RA patients
1	KThR	0.93	0.00	****	2.79	1.48	Elevated
2	KHR	0.91	0.00	****	2.57	1.36	Elevated
3	KVR	0.90	0.00	****	2.41	1.27	Elevated
4	Lysine	0.88	0.00	****	2.25	1.17	Elevated
5	Creatine	0.78	0.00	****	1.55	0.63	Elevated
6	GHR	0.78	0.00	****	1.27	0.34	Elevated
7	SHR	0.76	0.00	****	1.53	0.61	Elevated
8	Mannose	0.75	0.00	****	1.40	0.49	Elevated
9	3-HB	0.75	0.00	****	2.10	1.07	Elevated
10	Alanine	0.72	0.00	****	0.78	−0.35	Decreased
11	PTR	0.71	0.00	****	1.28	0.36	Elevated
12	Succinate	0.69	0.00	***	1.34	0.42	Elevated
13	Tyrosine	0.67	0.00	**	0.84	−0.26	Decreased
14	Urea	0.65	0.04	*	1.23	0.30	Elevated
15	Threonine	0.65	0.01	*	0.83	−0.27	Decreased
16	Acetate	0.63	0.01	**	0.79	−0.34	Decreased
17	Glutamate	0.63	0.02	*	0.75	−0.41	Decreased
18	Glycerol	0.63	0.71	NS	1.06	0.09	–
19	Glycine	0.62	0.06	*NS	1.13	0.18	Elevated
20	Histidine	0.61	0.04	*	0.90	−0.16	Decreased
21	Glycero-phosphocholine	0.61	0.05	*	1.25	0.32	Elevated
22	Glucose	0.60	0.12	NS	1.13	0.17	–
23	Isoleucine	0.60	0.03	*	0.86	−0.22	Decreased
24	Phenylalanine	0.59	0.24	NS	1.07	0.10	–
25	Leucine	0.59	0.05	*	0.85	−0.23	Decreased
26	QGR	0.58	0.23	NS	0.92	−0.12	–
27	Citrate	0.57	0.25	NS	0.82	−0.29	–
28	Acetone	0.57	0.02	*	1.52	0.60	Elevated
29	Betaine	0.55	0.57	NS	0.95	−0.07	–
30	Arginine	0.52	0.92	NS	0.99	−0.02	–
31	Pyruvate	0.52	0.91	NS	0.99	−0.02	–
32	Proline	0.52	0.77	NS	0.98	−0.03	–
33	Choline	0.52	0.19	NS	0.75	−0.42	–
34	Lactate	0.51	0.54	NS	0.96	−0.06	–
35	Valine	0.51	0.28	NS	0.94	−0.09	–
36	myo-Inositol	0.51	0.52	NS	0.95	−0.08	–
37	Dimethylsolfone	0.51	0.73	NS	1.04	0.06	–
38	Creatinine	0.51	0.54	NS	0.95	−0.07	–
39	Glutamine	0.50	0.91	NS	1.01	0.01	–

**Note:** Abbreviations used are: GHR: Glycine-to-Histidine ratio; KHR: Lysine-to-Histidine ratio; KVR: Lysine-to-Valine ratio (KVR), KThR: Lysine-to-Threonine ratio; PTR: Phenylalanine-to-Tyrosine ratio; SHR: Succinate-to-Histidine ratio; QGR: Glutamine-to-glucose ratio (QGR). The fold change (FC) is calculated following the mathematical process; FC: is real number, FC > 0; log2(FC) = y and FC = 2^y^. Note, the horizontal axis in volcano plot (Fig. 2D) represents log2(FC).

### 3.2 The diagnostic potential of discriminatory metabolites in RA

**Fig. 3:**
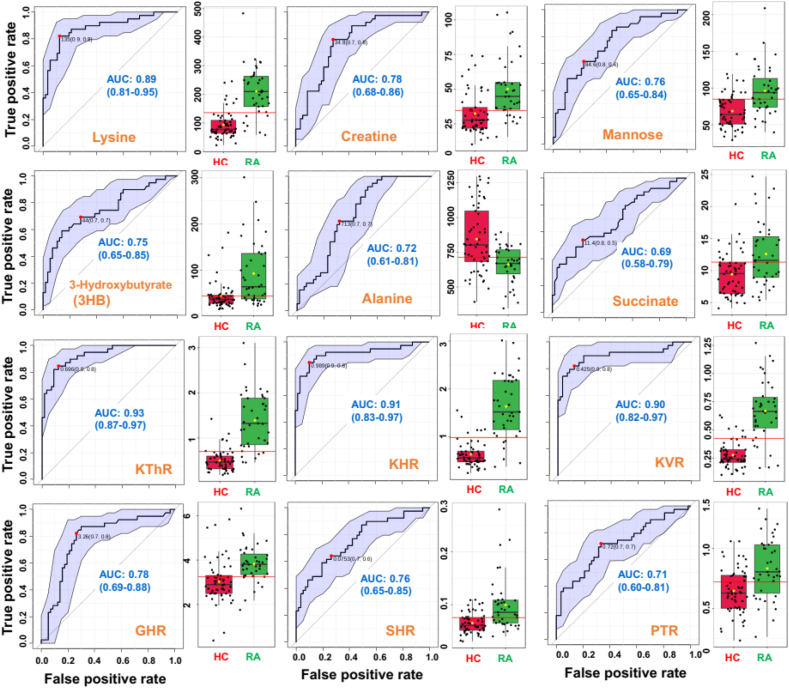
Top six discriminatory serum metabolic features and top 6 discriminatory serum metabolic ratios identified based on ROC curve analysis for comparison between RA patients and HC subjects. The computed 95% confidence interval (CI) for individual marker metabolites is highlighted in the faint blue background over the ROC curve. The area under the receiver operating characteristic curve (AUROC) values shown in red highlight the diagnostic potential of corresponding circulatory metabolite. The box-cum-whisker plots shown on the right side of each ROC curve plot shows the relative changes in the circulatory levels of these metabolites in the RA patients (in green) compared to HC (in red).

For evaluating the diagnostic potential of the discriminatory serum metabolites identified using OPLS-DA analysis (Fig. 2C), the receiver operating characteristic curves (ROC curves) were generated (Fig. 3) and the area under the ROC curve (AUC) were calculated for each discriminatory feature [33]. The AUC value < 0.5 indicates that the test feature has no diagnostic value, AUC of 0.65–0.8 indicates that the test feature has moderate accuracy, AUC of 0.8–0.9 indicates that the test feature has good accuracy, and AUC >0.9 indicates that the diagnostic test has high accuracy [18,34]. The ROC curves were generated for the top 20 discriminatory serum metabolites of which six metabolites (Lysine, mannose, creatine, 3-HB, Alanine and Succinate) were found with AUROC value > 0.67, suggesting these NMR based quantitative levels of serum metabolites exhibit moderate-to-good diagnostic potential for differentiating RA patients from HC cohort (the explicit AUROC values and corresponding statistical *t*-test parameters are listed in Table 8). The representative ROC curve plots of top six metabolites are shown in Fig. 3.

Ratiometric markers are promising when it comes to identification of biomarkers for a disease, predicting its severity, prognosis and monitoring treatment response [23,[25], [26], [27], [28], [29], [30]]. The present study used serum metabolite “formate” as an internal reference (with its concentration set at 0.03 mM) employing NMR suite of commercial software CHENOMX. The approach is similar to that followed for urinary metabolic profiling considering creatinine as an internal reference standard [1,2]. Therefore, the quantitative levels of other metabolites represent metabolite-to-formate ratio which may be influenced by formate concentration varying sample to sample. Therefore, it is expected that some serum metabolites may fail to show statistically significant change as demonstrated previously in NMR based clinical metabolomics studies from our lab [[3], [4], [5], [6]]. Therefore, a small exercise was further made to identify some relevant ratiometric features (metabolic ratios) of diagnostic potential using the biomarker module of Metaboanalyst 5.0 (a web based metabolomics data processing tool); Glycine to Histidine ratio (GHR, elevated levels are associated with active RA disease in preclinical rat models [31,32]), Lysine to Histidine ratio (KHR), Lysine to Valine ratio (KVR), Lysine to Threonine ratio (KThR), Phenylalanine-to-Tyrosine ratio (PTR, elevated levels are indicative of oxidative stress [27,28]), Succinate-to-Histidine ratio (SHR) and glutamine-to-glucose ratio (QGR, decreased levels are indicative of active autoimmune disease [25]). Finally, all the serum metabolic features (32 serum metabolic profiles and 7 metabolic ratios) were tested for their statistically significant change between the study groups employing volcano plot statistics approach. Clearly evident from the volcano plot that, out of seven metabolic ratios, six metabolic ratios (KVR, KHR, KThR, SHR, GHR and PTR) are significantly elevated in RA patients. The ROC curves generated for these significantly altered metabolic ratios are also shown in Fig. 3. Of them, three metabolic ratios (KThR, KHR and KVR) achieved good AUC values (>0.9) whereas 3 metabolic ratio (PTR, GHR and SHR) achieved fair AUC values (>0.7).

#### 3.2.1 Effect of whole system ayurveda intervention in rheumatoid arthritis

**Fig. 4:**
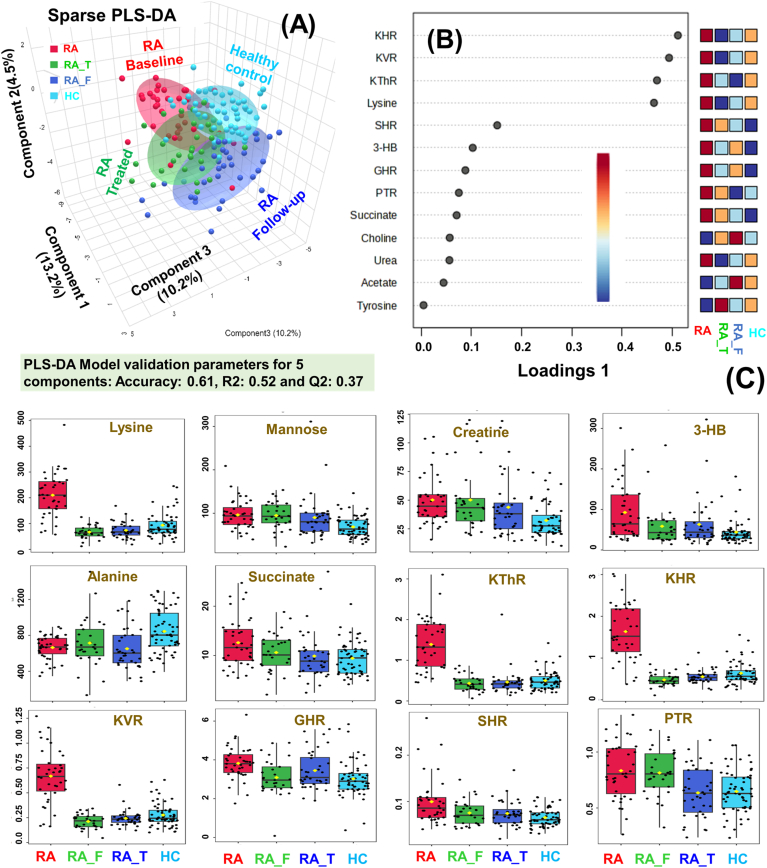
Sparse PLS-DA model-based analysis performed between study groups: RA (n = 37), RAF (n = 26), RAT (n = 36) and healthy control (HC, n = 57). **(A)** sPLS-DA score plot showing metabolic shifting towards normalization in RA patients after whole system ayurvedic treatment. The model validation of PLS-DA model (R2Y = 0.52; Q2 = 0.37) based on 39 metabolic features (32 metabolic profiles and 7 metabolic ratios). **(B)** Loading plot derived from sPLS-DA analysis in Metaboanalyst software highlighting the metabolites of discriminatory significance. **(C)** Representative box-cum-whisker plots showing quantitative variations of significantly altered serum metabolites and metabolic ratio between study groups: RA (n = 37), RAF (n = 26), RAT (n = 36) and healthy control (HC, n = 57). In each plot, the boxes denote interquartile ranges, horizontal line inside the box denote the median, and bottom and top boundaries of boxes are 25th and 75th percentiles, respectively. Lower and upper whiskers are 5th and 95th percentiles, respectively.

The sparse PLS-DA (sPLS-DA) algorithm produces robust and easy-to-interpret multivariate models for predictive monitoring of treatment response through reducing the number of variables (metabolites). Fig. 4A shows the 2D sPLS-DA score plot comparing the serum metabolic profiles of four study groups: RA (n = 37), RAF (n = 26), RAT (n = 36) and healthy control (HC, n = 57). The variance of RA baseline group is aligned along the first component and is perpendicular to the variance of HC group suggesting that RA and HC groups are clearly distinct. The overlap of RA_T and RA_F study groups with HC group was observed after three months of Ayurvedic whole system intervention. Further, the variance for HC, RA_T and RA_F groups were aligned perpendicular to the variance of baseline RA group further suggested that Ayurvedic whole system intervention for 3 months could reverse abnormalities in levels of specific metabolites (such as lysine, mannose, creatine, 3-HB, alanine and succinate) associated with pathology of RA. The relative metabolic changes in four study groups are clearly evident from the loading plot shown in Fig. 4B and from the representative box plots shown in Fig. 4C. The box plots in Fig. 4C have been derived from multi-group comparison based on ANOVA statistics performed using Metaboanalyst. The circulatory levels of metabolites are significantly different (as assessed based on the p-value <0.05) between study groups: baseline RA patients (in red), six-week follow-up RA patients after treatment (RA_F, in green), RA patients after three months of AWS treatment (RA_T, in blue) and healthy control (HC, in cyan) subjects.

## 4 Discussion

### 4.1 Effect of whole system ayurveda intervention in rheumatoid arthritis patients

There had been observation about effectiveness of single formulations or a combination of Ayurvedic formulations in RA. Various case reports and clinical trials of various strengths are available as published literature. The efficacy of Ayurvedic treatment for rheumatoid arthritis was observed through a cross-sectional experiential longitudinal study [35]. A Cochrane protocol for systemic review of Ayurvedic interventions in RA has also been published [36]. A systematic review of Ayurvedic medicine for rheumatoid arthritis conducted in 2005 observed a paucity of RCTs of Ayurvedic medicines for RA. The existing RCTs have not shown the strength that such treatments are effective therapeutic options for RA [37]. A Double-blind, randomized, controlled, pilot study comparing classic ayurvedic medicine, methotrexate, and their combination in rheumatoid arthritis, which was a first-ever study comparing Ayurveda, MTX, and their combination, found all 3 treatments approximately similar in efficacy, within the limits of a pilot study. Adverse events were however numerically fewer in the Ayurveda-only group. This study demonstrated that double-blind, placebo-controlled, randomized studies are possible when testing individualized classic Ayurvedic versus allopathic treatment in ways acceptable to western standards and to Ayurvedic physicians [38]. In general, current literature and clinical trials have not been convincing enough to show the efficacy of Ayurvedic interventions in RA. In this scenario, this pilot study which at one point observed the clinical changes in the study participants receiving the whole system Ayurveda interventions and on the other has examined the metabolomics profile of the patients on pre and post treatment basis and its comparison with healthy controls, has come as a definitive advancement upon the current state of knowledge. The clinical observation has shown statistically and clinically significant reductions on the parameters like SJC, TJC, DAS-28 ESR and PGHA. Ayurvedic parameter of AMA score has also shown a significant post treatment reduction in such patients receiving the Ayurveda interventions for 3 months.

## 5 Effect of treatment revealed by metabolic profiles

**Fig. 5:**
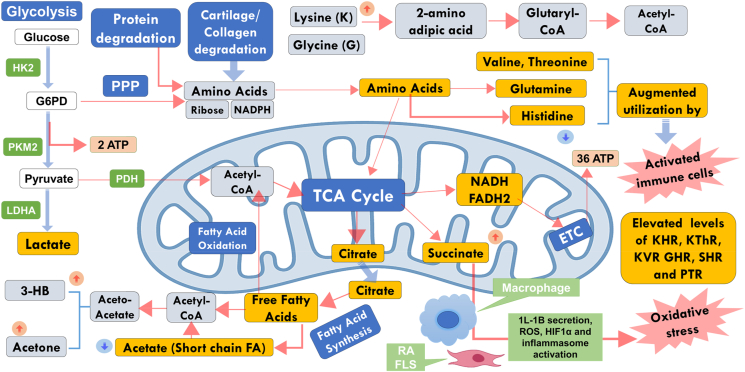
Schematic showing altered metabolic pathways in synovial joints of RA patients which are reflecting their impact on the blood circulatory system. The abbreviations used are: PPP: Pentose Phosphate Pathway; HK2: Hexokinase; PKM2: Pyruvate Kinase M2; PDH: Pyruvate dehydrogenase; FLS: Fibroblast-like synoviocytes; PTR: Phenylalanine-to-tyrosine ratio; SHR: Succinate-to-histidine ratio; KHR: Lysine-to-histidine ratio; GHR: Glycine-to-histidine ratio; FA: Fatty Acid; 3-HB: 3_hydroxybutyrate; ATP: Adenosine tri-phosphate; TCA: Tricarboxylic acid; NADH: Nicotinamide adenine dinucleotide; NADPH: NADH phosphate; FADH2: Flavin adenine dinucleotide.

Rheumatoid arthritis (RA) is a chronic inflammatory disease that causes joint inflammation, bone and cartilage destruction, and sometimes disability. The pathophysiology of RA is multifactorial and, to a significant extent, unknown. The serum metabolomics analysis revealed the altered levels of several metabolites such as glycine, tyrosine, 3HB, acetate, acetone and some essential amino acids such as lysine, histidine, phenylalanine, threonine, and valine. The succinct summary of key metabolic changes driving the pathophysiology of RA is schematically depicted in Fig. 5 and exquisitely discussed further.

Abnormal glycine metabolism is well established pathophysiological feature in RA [39] and the circulatory levels of glycine are largely reported to be elevated in RA [40]. Further, various clinical studies report the decreased circulatory levels of histidine in RA Patients. The low free serum histidine levels have also been found to be associated with disease activity in RA [41]. Other studies reported higher levels of free histidine in the synovial fluid (SF) compared to that in the patient serum samples of RA but SF histidine levels were significantly lower than corresponding results in patients with osteoarthritis [42]. Therefore, we hypothesized and demonstrated that the circulatory glycine to histidine ratio (GHR) is significantly elevated in RA compared to HC (with p-value <0.05) and its circulatory levels decrease towards the normal circulatory levels after the Ayurvedic treatment (Fig. 5).

RA is associated with mitochondrial dysfunction in various cell types, including immune cells and synovial fibroblasts. Mitochondrial dysfunction leads to increased succinate production and altered TCA cycle flux. Succinate has also been recognized as a signalling molecule impacting the immune function. In the context of RA, immune cells such as macrophages and T cells are central players in the disease pathogenesis. Succinate is shown to influence the activation and polarization of macrophages [43]. Elevated succinate levels can promote the pro-inflammatory M1 phenotype of macrophages, contributing to the chronic inflammation observed in RA. Consistent with this pathophysiological phenomenon, the increased circulatory levels of succinate may contribute to oxidative stress and inflammation observed in RA (Fig. 5). This study clearly reveal that the Ayurvedic treatment is lowering the circulatory levels of succinate in the sera of RA patients and after the completion of the Ayurvedic treatment, the succinate levels are decreased to the levels observed in HC subjects (Fig. 2B). Further, the study demonstrated that the circulatory succinate-to-histidine ratio (SHR) is significantly elevated in RA compared to HC (with p-value <0.05) and its circulatory levels decrease towards the normal circulatory levels after the Ayurvedic treatment (Fig. 4B).

RA can lead to muscle wasting and weakness due to factors like chronic inflammation and immune system dysregulation. Immune cells involved in inflammation require energy to perform their functions. Creatine plays a vital role in the production of adenosine triphosphate (ATP) [44]. The availability of ATP, which creatine contributes to producing, could impact immune cell activity and inflammation levels in RA. Elevated creatine levels might be a reflection of elevated inflammation and oxidative stress in RA patients leading to muscle wasting and joint damage. The study reveals that the Ayurvedic treatment is lowering the circulatory levels of creatine in the sera of RA patients and after the completion of the Ayurvedic treatment, the creatine levels are decreased to the levels observed in HC subjects (Fig. 4B).

The present study also revealed that the circulatory levels of mannose are significantly elevated in RA compared to HC (with p-value <0.05) and its circulatory levels decrease towards the normal circulatory levels after the Ayurvedic treatment (Fig. 4B). Mannose is a component of glycoproteins and glycolipids found on the surface of cells and plays a role in several biological processes, including cell communication, immune response regulation, and energy metabolism [45]. Glycosylation, the process of attaching sugar molecules like mannose to proteins and lipids, is crucial for immune system function. Glycosylation patterns on immune cells and molecules can influence immune responses. The elevated mannose levels might be related to abnormal glycosylation and so altered cellular signalling cascades that contribute to inflammatory responses in RA.

The potential hallmark of this metabolomics study is the markedly increased circulatory levels of lysine and lysine-to-histidine ratio (KHR), Lysine-to-valine ratio (KVR), Lysine-to-Threonine ratio (KThR) in the sera of RA patients (Fig. 4A). These results are well consistent to previous metabolomics studies showing decreased circulatory levels of histidine and valine in the sera of RA patients [46].

Lysine (a non-glycogenic amino acid) plays several vital roles in biological processes including protein synthesis, collagen formation, immune functioning, calcium absorption, nitrogen balance, supporting wound healing and maintaining tissue integrity [47]. Of them, the collagen protein forms the structural framework of connective tissues such as skin, cartilage, and tendons. Lysine is critical for collagen formation due to its involvement in cross-linking collagen molecules, contributing to the stability, structure, and functionality of various connective tissues in the body. The elevated lysine levels in the serum samples of RA patients might be related to collagen degradation and its impaired degradation pathways [48]. Collagen degradation in rheumatoid arthritis (RA) is a complex process involving the interplay of inflammatory cytokines, proteolytic enzymes (such as cathepsins, Matrix Metalloproteinases (MMPs), autoimmune responses, and cartilage breakdown. Further, the availability of lysine may interfere with the epigenetic regulation and thus may influence gene expression patterns and contribute to the epigenetic landscape in rheumatoid arthritis. In RA, joint damage and degradation of cartilage are key features which might be the reason for elevated circulatory levels of lysine in RA patients. The present study clearly revealed that the Ayurvedic treatment is lowering the circulatory levels of lysine in the sera of RA patients and after the completion of the Ayurvedic treatment, the lysine levels are decreased to the levels observed in healthy controls (Fig. 4B). Therefore, targeting pathways involved in collagen degradation is a key therapeutic strategy in managing RA to prevent joint damage and preserve joint function.

## 6 Conclusion

Ayurvedic system of medicine enjoys the trust of people for variety of clinical conditions. Arthritis is one prominent clinical condition often sought by people for Ayurvedic interventions. Although patients and physician both enjoy the benefits of Ayurveda in these condition, dearth of rigorous clinical trials has made it hard for others to believe it. Doing RCTs which are considered gold standards from the perspectives of a clinical study, has its own limitations when it is adopted for Ayurveda, especially to its whole system intervention composed of a complex regime to treat a patient. Some recent attempts to do the RCTs in Ayurveda with a whole system intervention are still far from the conventional research practices in Ayurveda. In this situation, metabolomics studies have come up as an additional tool to support the observations made during the clinical study by finding fine metabolomics changes observed after the interventions. The present NMR based clinical metabolomics study exquisitely demonstrated the intervention effect of Ayurvedic whole system intervention on Rheumatoid Arthritis (RA) patients. The study assessed the clinical outcomes as well as changes in metabolomics profiles of RA patients compared to healthy controls. The significant clinical improvements observed in RA patients following the Ayurveda whole system intervention include a reduction in the Disease Activity Score-28 Erythrocyte Sedimentation Rate (DAS-28 ESR), a decrease in the Ayurvedic Ama Score (AMA Score), a reduction in the total number of swollen joints (SJC), and a decrease in the total number of tender joints (TJC). These improvements indicate a lessening of disease activity and inflammation in the patients who received the Ayurvedic intervention. Further, the study compared the pre- and post-treatment metabolomics signatures of these patients with respect to healthy control (HC) subjects to identify metabolites and metabolic ratios that differ significantly between RA patients and healthy individuals and if these metabolic changes improve with treatment. Compared to healthy subjects, six circulatory metabolites and 6 metabolic ratios were significantly altered in RA patients compared to healthy controls. These included increased levels of succinate, lysine, mannose, creatine, and 3-hydroxybutyrate and decreased levels of alanine in RA patients, which shifted towards healthy control levels post-treatment.

In conclusion, this study highlights the potential of metabolomics studies as a potential tool (a) to substantiate clinical observations made in Ayurveda related clinical trials and (b) to improve the understanding the mechanism of action of Ayurvedic interventions in RA. While this study provides a robust foundation for the therapeutic benefits of AWS intervention in RA, further research is imperative to validate these preliminary findings and to elucidate the precise mechanisms by which Ayurvedic treatments exert their beneficial effects. Such investigations will not only enhance our understanding of the complex interplay between Ayurvedic medicine and human metabolism but also pave the way for the development of more targeted and effective Ayurvedic therapies for RA and other inflammatory conditions.

## Sources of funding

This research work was partly supported under the intramural funding from the Centre of Biomedical Research (CBMR), Lucknow (Project No. CBMR/IMR/0010/2021).

## Data availability statement

The NMR data is available without undue reservation for further studies on request to the corresponding authors.

## Author contributions

**SR**: Conceptualization, Methodology, Writing- Original Draft Preparation. Writing - Review & Editing **AV**: Investigation. **RT**: Visualization, Investigation. Formal analysis. **AS**: Investigation. Formal analysis Writing - Review & Editing: **DK**: Conceptualization, Data analysis, Writing - Review & Editing, Supervision, Software, Validation**.**

## Declaration of generative AI in scientific writing

We state that any help from generative AI or AI assisted technology has not been obtained in writing of this manuscript.

## Declaration of competing interest

On behalf of all authors, the corresponding authors state that there is no conflict of interest. The authors declare that they have no known competing financial interests or personal relationships that could have appeared to influence the work reported in this paper.
